# The impact of dysmenorrhea on psychological wellbeing and academic achievement among Jordanian female university students

**DOI:** 10.3389/fgwh.2026.1776436

**Published:** 2026-06-10

**Authors:** Arwa Alsaraireh, Anoud Alblewi, Ishraq Al-Sarairhe, Mohammed Qutishat

**Affiliations:** 1 Department of Maternal and Child Health Nursing, Faculty of Nursing, Mutah University, Al-Karak, Jordan; 2 Faculty of Nursing, Al-Zaytoonah University of Jordan, Amman, Jordan; 3 Faculty of Nursing, Jerash University, Jerash, Jordan; 4 Community and Mental Health Department, College of Nursing, Sultan Qaboos University, Muscat, Oman

**Keywords:** academic performance, Jordan, primary dysmenorrhea, psychological health, university students

## Abstract

**Background:**

Primary dysmenorrhea (PD) is one of the most common gynecological conditions among young women. It is characterized by menstrual pain in the absence of pelvic pathology. However, little research has been conducted on its psychological and academic impact, particularly among female university students. This study aims to determine the relationship between primary dysmenorrhea and academic performance as well as mental health in a sample of female university students in Jordan.

**Methods:**

An online, cross-sectional survey was conducted between August and November 2025. A total of 492 female university students at Mutah University, Jordan, completed a self-structured questionnaire on sociodemographic and menstrual history. The severity of PD was assessed using the WaLIDD (Working ability, Location, Intensity, Days of Pain) scale, while psychological wellbeing was assessed using the Depression, Anxiety, and Stress Scale (DASS-21). Academic performance was assessing using self-reported cumulative grade point average. Descriptive and inferential statistics were used to analyze the data, with a significance level of *p* < 0.05.

**Results:**

Participants were predominantly (91.1%) between the ages of 18 and 24, single (84.8%), and Jordanians (94.7%). PD was reported by the majority, with 73.8% reporting pain lasting 1–2 days, and 43.3% skipping classes due to menstrual pain. Age, sleep duration, education level, dietary habits, analgesic use, pain duration, and family history of dysmenorrhea significantly predicted PD severity (final model *R*² = 25.4%, *p* < 0.001). Mean DASS-21 scores indicated mild levels of depression (*M* = 1.11, SD = 1.55), anxiety (*M* = 1.15, SD = 1.38), and stress (*M* = 0.82, SD = 1.21).

**Conclusion:**

Primary dysmenorrhea is highly prevalent and has an impact on academic achievement among Jordanian female university students. The identified predictors offer targeted interventions and highlight the need for in-campus health services to improve students' menstrual health and academic outcomes.

## 1 Introduction

Even though menstruation is a normal and natural part of the female monthly cycle, many women do not view it as a normal or comfortable occurrence (1). Many women experience pain during their menstrual cycle. This condition is known medically as dysmenorrhea. This term describes the recurrent cramp-like pain that comes with menstruation (2). In addition to being widespread, dysmenorrhea is very debilitating, impacting school attendance, work performance, social involvement, and overall quality of life (3).

Dysmenorrhea is generally divided into two types: primary or secondary (2). Primary dysmenorrhea (PD) refers to menstrual pain that occurs in the absence of pelvic disorder (4). It is commonly seen in adolescent and young women, with many reporting that the discomfort begins within the first year or two after menarche (5). In contrast, secondary dysmenorrhea (SD) is linked to gynecological conditions. Some pain and symptom relief may occur during the first couple of days of menstruation (6). However, the pain often radiates from the pelvis to the lower back or legs and may be accompanied by nausea, vomiting, headache, dizziness, fatigue, and sleep disturbances (6). Such symptoms can interfere with daily activities, concentration, social participation, and overall confidence (3).

Prevalence rates of PD vary widely across countries, influenced by variation in assessment tools, cultural perceptions of pain, and research methodology. In addition, prevalence reports may be influenced by self-report bias. Recent studies (between 2019 and 2025) have indicated that PD is highly common among young women worldwide. For instance, research from Croatia reported a prevalence of 90.1% (7), while research from Pakistan reported an even higher prevalence of 92.7% (7). Nigeria has one of the highest rates in the world at 96.5% (8), whereas Lebanon (9) and Saudi Arabia (10) reported a prevalence of 80.9%.

A recently published systematic review and meta-analysis (11) documented a high prevalence of menstrual disorders among nursing students. Dysmenorrhea was reported at a prevalence of approximately 72%, while premenstrual syndrome and premenstrual dysphoric disorder (PMDD) were recorded at 62% and 21%, respectively. The study further demonstrated negative consequences, including poor academic achievement due to absenteeism and difficulty concentrating in class, which negatively affected students' quality of life (11). Another systematic review and meta-analysis analyzed the effects of menstrual abnormalities on academic and sociological aspects among female medical students worldwide. It reported a prevalence rate of approximately 72.7% for dysmenorrhea, along with high rates for premenstrual syndrome and premenstrual dysphoric disorder. One important conclusion is that menstrual abnormalities are widespread and are significantly associated with poor academic achievement, including absence from classes, reduced concentration, and decreased quality of life (12).

The severity of PD also varies among women (7). While mild symptoms are often manageable, moderate-to-severe PD can significantly disrupt daily function and cause mental distress (5, 13). Several factors have been associated with more severe pain, including stress, family history of dysmenorrhea, lifestyle habits, individual pain sensitivity, and the number of years since menarche. When not properly managed, PD can negatively affect academic performance and contribute to psychological stress (3).

Mood changes are common during menstruation, making psychological wellbeing an important factor in coping with dysmenorrhea. Research indicates that women often experience sleep problems, difficulty concentrating, and emotional instability, all of which affect academic performance (5, 14). Moreover, research suggests that mental health problems, such as anxiety, sadness, and stress, can exacerbate dysmenorrhea through inflammatory processes, hormonal interactions, and stress pathways (15). Menstrual pain is intensified by psychological discomfort, while psychological symptoms are worsened by severe menstrual pain, suggesting a bidirectional relationship (8).

Several studies support this association. According to research by Adib-Rad and colleagues (16), female students with dysmenorrhea, especially those with a family history of dysmenorrhea and lower maternal education, experienced higher psychological discomfort. Similarly, a study among Jordanian medical students (17) reported a strong correlation between increased dysmenorrhea severity and COVID-19-related mental health symptoms. In contrast, research among Pakistani university students found that even though most of them had PD and faced considerable academic disruptions, there were no significant associations between PD and mental health problems (7). These varied results underscore the need for further research using standardized methods across diverse demographics.

Female university students may be more vulnerable, since academic pressure can increase psychological discomfort, which can aggravate PD (7). Despite the high prevalence of PD among young women, there is little information in Jordan about its combined consequences on psychological health and academic performance among university students. Understanding PD and its impact on psychological and academic outcomes is important for developing targeted interventions to improve students' wellbeing, menstrual health, and academic achievement. Therefore, this study aimed to investigate the prevalence, severity, and associated factors of primary dysmenorrhea among female university students in Jordan, and to assess its impact on psychological wellbeing and academic performance.

## 2 Material and methods

### 2.1 Study design and setting

A descriptive cross-sectional correlational design was employed between August and November 2025. This study was conducted at Mutah University, one of the largest public universities in Al-Karak, Jordan. Mutah University is a public university established by Royal Decree on 22 March 1981, serving as a national institution of higher education for both military and civilian students. The university comprises 15 colleges: seven scientific colleges, seven humanities colleges, and the College of Graduate Studies. In 2025, Mutah University had a total student population of 21,918, comprising 12,442 female students (56.8%) and 9,476 male students (43.2%).

### 2.2 Study sample

Participants were selected through convenience sampling. The study sample included female students aged 18 and older who were actively enrolled at Mutah University and had regular menstrual cycles. Participants were excluded if they had secondary dysmenorrhea, PMDD, gynecological problems (e.g., polycystic ovarian syndrome), systemic illnesses, a significant surgical history, pregnancy, or nursing. This exclusion was due to our study's focus on primary dysmenorrhea. Informed consent was obtained electronically via consent declaration located on the first page of the online survey.

### 2.3 Sample size

Slovin's formula was used to calculate the sample size (*n*), based on the population size (*N*) and the margin of error (*e*). It was computed as explained in previous work (18). Based on Slovin's formula, a minimum of 383 participants were sufficient to achieve a 95% confidence level and a 5% margin of error; however, the accessible population consisted of 12,442 female university students, with 5,705 from scientific colleges, 5,534 from humanities and social sciences, and 1,203 from the College of Graduate Studies. With a predicted attrition rate of 20%, a minimum sample size of 466 was determined. The sample size was increased to 492 to compensate for the non-responses or incomplete responses.

### 2.4 Data collection

Data were collected using a self-administered online questionnaire. The survey was distributed in Arabic and reviewed by experts in women's health to ensure clarity, content validity, and cultural appropriateness. Before data collection, all participants were informed of their right to withdraw at any time during the study and that their participation was voluntary, as stated in the information provided on the questionnaire cover sheet. The questionnaire was distributed electronically via Microsoft Forms in Teams during online lectures, particularly lectures on elective subjects with many students from various faculties and specializations, after obtaining permission from the lecturer.

Participants who completed the survey and did not withdraw were considered to have given their consent, affirming their voluntary participation. In online questionnaires, participants are asked to click the “I agree to participate” option, which appeared as the first question. To facilitate communication and address any questions from study participants, a researcher's contact information, including phone number and email, was provided in the information sheet.

### 2.6 Study instrument

The study questionnaire comprised four sections assessing sociodemographic variables, menstrual health, psychological wellbeing, and academic success. The questionnaire was written in English, then translated into Arabic, and then back-translated into English for validation.

Part 1 of the questionnaire collected sociodemographic information and menstrual characteristics. This included participants' age, nationality, marital status, family history of dysmenorrhea, chronic medical condition, smoking habits, habitual caffeine consumption, daily sleep duration, maternal education level, participants' education level, academic year, college affiliation, weight, height, and calculated body mass index (BMI). Variables associated with menstruation included age at menarche, cycle regularity, duration, frequency, intensity, and the number of sanitary pads used per cycle.

Part 2 assessed PD using the WaLIDD scale (Working Ability, Location, Intensity, Days of Pain) (19). Permission to use the tool was obtained from the original author. The scale comprises four items: pain location, pain intensity assessed via the Wong–Baker Pain Scale, number of days of menstrual pain, and frequency of pain interfering with daily activities during the three preceding cycles. Each item is scored from 0 to 3, yielding a total score ranging from 0 to 12, with higher scores indicating more severe dysmenorrhea. Dysmenorrheal severity is categorized as follows: 0 = no dysmenorrhea; 1–4 = mild; 5–7 = moderate; and 8–12 = severe. The WaLIDD scale demonstrated acceptable reliability (Cronbach's *α* = 0.723) and validity in previous studies (19), and it has also been validated among Arabic-speaking populations, demonstrating good psychometric properties (20). In the present study, the internal reliability was good (Cronbach's *α* = 0.86). The Wong–Baker Pain Scale is a validated measure of pain intensity and was adapted to a four-level scale for use within this instrument.

Part 3 assessed psychological wellbeing using the Depression, Anxiety, and Stress Scale—21 items (DASS-21) (21). DASS-21 evaluates three related negative emotional states: depression, anxiety, and stress. Each subscale contains seven items: DASS-Depression (items 3, 5, 10, 13, 16, 17, 21), DASS-Anxiety (items 2, 4, 7, 9, 15, 19, 20), and DASS-Stress (items 1, 6, 8, 11, 12, 14, 18). Responses are scored on a four-point Likert scale ranging from zero (“did not apply to me at all”) to 3 (“applied to me very much or most of the time”). Severity thresholds for depression, anxiety, and stress were based on established cutoffs (21). In the present study, internal consistency was excellent, with Cronbach's alpha values of 0.84 for depression, 0.83 for anxiety, and 0.83 for stress. The Arabic version of DASS-21 has been widely used and validated in Arabic-speaking populations, with previous studies confirming its internal consistency and construct validity (22, 23).

Part 4 assessed academic performance using Mutah University's formally adopted cumulative grade-based methodology. Participants self-reported their cumulative grade point average (CGPA) by selecting the appropriate score range and grade. Academic achievement was divided into four categories: excellent (84–100), very good (76–83.99), good (68–75.99), and acceptable (60–67.99). This approach ensured that academic progress was measured in a standardized, consistent, and contextually appropriate manner, in accordance with the university's official evaluation criteria.

### 2.7 Ethical considerations

Ethical approval for this study was obtained from the Ethics and Research Committee at the Faculty of Nursing, Mutah University (Ref No. EC9/2025). Informed consent was obtained from all participants prior to data collection. Participants were clearly informed that participation was voluntary, that they had the right to refuse or withdraw at any time without penalty or loss of benefits, and that their decision would not affect their academic standing or any related services. Confidentiality and anonymity were strictly maintained throughout the study. No names or direct personal identifiers were collected on the questionnaire. All collected data were handled confidentially and stored securely on a password-protected personal computer accessible only to the principal investigator.

### 2.8 Statistical analysis

Data were processed using IBM SPSS Statistics version 28. Descriptive statistics, such as frequencies, percentages, means, and standard deviations, were used to summarize participants' sociodemographic characteristics, educational background, lifestyle factors, menstruation-related variables, menstrual pain characteristics, psychological health scores (DASS-21), and academic performance.

The associations between menstrual pain severity and potential factors were investigated using hierarchical multiple regression. Five models were developed to determine the incremental contribution of various predictor sets to differences in menstrual pain severity.

- Model 1: This model included demographic variables (age, marital status, and nationality).
- Model 2: In this model, we incorporated participant educational information (academic year, college, student educational level, maternal education, and missed lectures) in addition to Model 1 predictors.
- Model 3: In addition to the predictors in models 1 and 2, this model added more variables related to women's lifestyle, including history of smoking, coffee intake, sleep duration, dietary patterns, and medical history.
- Model 4: To further investigate the predictors of menstrual pain severity, we added more predictors to the previous models related to women's menarche history, such as onset of menarche, family history of dysmenorrhea, menstrual cycle characteristics including frequency, duration, and intensity, and the number of sanitary pads used daily.
- Model 5: In the final model, in addition to all previous models, we added specific details describing women's pain during their cycle, such as the use of painkillers, pain location, pain severity, pain duration, impact on daily activities, gynecologist visits, and family history.

Prior to analysis, all multiple regression assumptions, including linearity, normality, and multicollinearity, were assessed to confirm the validity of the regression results. Models *R*, *R*², and adjusted *R*² values were used to present the variance in menstrual pain severity explained by predictors. For each predictor, *p*-value < 0.05 was considered significant, and analysis of variance (ANOVA) was used to determine the significance of each regression model (1–5).

## 3 Results

Participant characteristics are presented in Tables 1–3. The sample was predominantly young adults aged 18–24 years (91.1%), single (84.8%), and Jordanian (94.7%). Most participants were enrolled in bachelor's programs (95.5%), mainly in scientific colleges (74.8%), and the majority reported good to excellent academic performance.

**Table 1:** Participant demographics (*N* = 492).

Variable	*N*	%
Age		
18–24 years	448	91.1
25–29 years	35	7.1
30–39 years	9	1.8
Marital status		
Single	417	84.8
Married	72	14.6
Divorced	3	0.6
Nationality		
Jordanian	466	94.7
Others	26	5.3

**Table 2:** Participant educational information (*N* = 492).

Variable	*N*	%
Student level of education		
Bachelor of science in nursing	470	95.5
Graduate level	22	4.5
Academic year		
First year	58	11.8
Second year	96	19.5
Third year	175	35.6
Fourth year	163	33.1
Student college		
Humanities and social sciences	115	23.4
Scientific	368	74.8
Post-graduate studies	9	1.8
Cumulative grade point average		
Acceptable 60–67.99	18	3.7
Good 68–75.99	98	19.9
Very good 76–83.99	221	44.9
Excellent 84–100	155	31.5
Missed a lecture due to menstrual pain		
No	279	56.7
Yes	213	43.3

**Table 3:** Participant lifestyle (*N* = 492).

Variable	*N*	%
BMI		
<18.5 underweight	23	4.7
18.5–24.9 normal weight	53	10.8
25–29.9 overweight	305	62.0
>30 obese	111	22.6
Smoking		
No	469	95.3
Yes	23	4.7
Drinking coffee		
No	201	40.9
Yes	291	59.1
Sleeping hours		
Less 6 h	78	15.9
From 7–8 h	331	67.3
More 9 h	83	16.9
Eat sweets or chocolate		
Daily	109	22.2
A few times a week	256	52.0
Occasionally	84	17.1
Rarely	43	8.7
Chronic medical conditions		
No	483	98.2
Yes	9	1.8

Lifestyle characteristics indicated that 62% of participants were overweight. Most reported sleeping 7–8 h per night (67.3%), were non-smokers (95.3%), and consumed coffee regularly (59.1%). In addition, 52% reported eating sweets or chocolate a few times per week, while 98.2% reported no chronic medical conditions.

Characteristics of participants' menstrual history are presented in Table 4. Regarding menstrual characteristics, 55.1% experienced menarche at ages 12–13, and 77.2% reported regular menstrual cycles. Most participants reported cycle lengths of 20–31 days (81.3%) and duration of 5–7 days (75.0%), with 83.9% reporting moderate menstrual flow. Nearly half (47.4%) used 3–5 sanitary pads per day. In addition, 54.1% reported no family history of dysmenorrhea, while 24.4% reported a positive family history.

**Table 4:** Characteristics of participants' menstrual history.

Variable	*N*	%
Age of menarche		
From 9 to 11 years	54	11.0
From 12 to 13 years	271	55.1
From 14 years and older	167	33.9
Regularity of menstrual cycle		
Not regular	112	22.8
Regular	380	77.2
Frequency of the menstrual cycle		
Less than 20 days	24	4.9
From 20 to 31 days	400	81.3
More than 31 days	68	13.8
Duration of the menstrual cycle		
Less than 5 days	81	16.5
From 5 to 7 days	369	75.0
More than 7 days	42	8.5
Intensity of the menstrual cycle		
Light	44	8.9
Medium	413	83.9
High	35	7.1
Daily pads used during the menstrual period		
>3 pads	138	28.0
3–5 pads	233	47.4
6–7 pads	92	18.7
>7 pads	29	5.9
Family has a history of dysmenorrhea		
No	266	54.1
Yes	120	24.4
Not sure	106	21.5

A notable functional impact was observed, with 43.3% of participants missing lectures due to menstrual pain.

Menstrual pain was most commonly experienced in the lower abdomen (83.5%), with a small percentage reporting pain in the lumbar region (8.7%). About half of the participants (51.6%) reported using painkillers such as ibuprofen to manage menstrual pain. Regarding pain characteristics, 54.7% reported no or minimal pain, while 41.1% reported mild pain, and a smaller proportion reported moderate-to-severe pain. Most participants (73.8%) experienced pain lasting 1–2 days.

Notably, 48.6% of participants rarely experienced disturbance in daily activities, and only 3.5% reported visiting a gynecologist for menstrual pain management. Table 5 presents these findings in detail.

**Table 5:** Menstrual pain description (*N* = 492).

Variable	*N*	%
Use painkillers such as Ibuprofen to relieve menstrual pain		
No	238	48.4
Yes	254	51.6
Most frequent location of menstrual pain		
Lower limbs	14	2.8
Lumbar region	43	8.7
Low abdomen	411	83.5
Inguinal region	24	4.9
Severity of your menstrual pain		
Does not hurt	225	45.7
Hurt a little bit	202	41.1
Hurts more	42	8.5
Hurts even more	23	4.7
Pain duration		
0 days	25	5.1
1–2 days	363	73.8
3–4 days	104	21.1
Impact of pain on daily activity		
Never	162	32.9
Rarely	239	48.6
Always	91	18.5
Visited a gynecologist due to menstrual pain		
No	475	96.5
Yes	17	3.5
Family has a history of dysmenorrhea		
No	266	54.1
Yes	120	24.4
Not sure	106	21.5

The DASS-21 scores are presented in Table 6. The mean scores were as follows: depression subscale (*M* = 1.11, SD = 1.55), anxiety subscale (*M* = 1.15, SD = 1.38), and stress subscale (*M* = 0.82, SD = 1.21). These values represent mean item-level scores for each subscale rather than total raw scores. Based on standard DASS-21 scoring procedures, these results indicate relatively low levels of depression, anxiety, and stress among participants.

**Table 6:** Descriptive statistics of participants' DASS-21 (Depression, Anxiety, and Stress Scale-21) scores.

Variable	Range	Minimum	Maximum	Mean	Std. deviation
Depression subscale	7.00	0.00	7.00	1.11	1.55
Anxiety subscale	10.00	0.00	10.00	1.15	1.38
Stress subscale	7.00	0.00	7.00	0.82	1.21
Total DASS score	—	—	—	3.09	3.318

The results of the regression analyses provide a comprehensive understanding of the factors associated with menstrual pain severity (Table 7). In the first model (only demographic variables), the results for *R*, *R*^2^, and adjusted *R*^2^ were 0.043, 0.002, and −0.004, respectively, indicating negligible explanatory power, explaining less than 0.2% of the variance in pain severity.

**Table 7:** Multivariate regression analysis model.

Analysis	Model	Predictors included	*R*/*R*²/adjusted *R*²	Significant predictors (*p* < 0.05)	Key findings
Multiple regression	1	Participants demographics^a^	*R* = 0.043	None significant	The model explains very little variance in menstrual pain severity
			*R*² = 0.002		
			Adjusted *R*² = −0.004		
Multiple regression	2	Model 1 + Participant educational information^b^	*R* = 0.233	Age (*p* = 0.013)	Significant predictors include student educational level, age, and sleep hours
			*R*² = 0.054	Student education (*p* < 0.001)	
			Adjusted *R*² = 0.039	Daily sleeping hours (*p* = 0.012)	
Multiple regression	3	Models 1 and 2 + lifestyle^c^	*R* = 0.282	Age (*p* = 0.022)	Additional variables modestly improve model fit; significant factors include age, sleep, and dietary habits
			*R*² = 0.080	Student education (*p* < 0.001)	
			Adjusted *R*² = 0.053	Sleep hours (*p* = 0.012)	
				Eating sweets (*p* = 0.040)	
Multiple regression	4	Model 1, 2, and 3 + Participants menarche history^d^	*R* = 0.354	Intensity (*p* < 0.001)	The model further explains variance; significant predictors include age, sleep, medication use, and pain duration
			*R*² = 0.125	Regularity (*p* < 0.001)	
			Adjusted *R*² = 0.076	Pads (*p* = 0.033)	
				Family history (*p* = 0.002)	
Multiple regression	5	Model 1,2,3, and 4 + description of menstrual pain^e^	*R* = 0.504	Age (*p* = 0.005)	The final model explains 25.4% of the variance (*R*²), while the adjusted *R*² indicates that approximately 18.9% of the variance is explained after adjustment for model complexity
			*R*² = 25.4%	Sleep hours Severity (*p* < 0.001),	
			Adjusted *R*² = 18.9%	Location (*p* < 0.001)	
				Duration (*p* < 0.001)	
				Painkillers (*p* < 0.001)	
ANOVA (Model 4)	4	Same predictors as Model 4	*F* = 2.556 *p* < 0.001	Overall significant model	The full model significantly predicts menstrual pain severity

a Demographics: nationality, age, marital status.

b Participant Educational information: missed lectures, colleges, mother's education, academic year, student education.

c Lifestyle: Eating sweets, medical history, coffee, BMI, smoking, age at menarche, menstrual cycle variables.

d Participants' menarche history: age of menarche, regularity of menstrual cycle, frequency of the menstrual cycle, duration of the menstrual cycle, intensity of the menstrual cycle, daily pad usage during menstrual period, family has a history of dysmenorrhea.

e Participants' description of menstrual pain: use painkillers, location of menstrual pain, severity of pain, pain duration, pain impact, visited a gynecologist, dysmenorrhea family history.

In the second model, which included participants' educational information, the model yielded an *R* of 0.233 and an *R*^2^ of 0.054, explaining approximately 5.4% of the variance in menstrual pain severity. This increase was associated with three significant predictors—age (*p* = 0.013), student educational level (*p* < 0.001), and daily sleep duration (*p* = 0.012)—indicating that menstrual pain severity increased with older age, higher educational attainment, and longer sleep durations.

In model three, where lifestyle factors were added, the result indicated a modest enhancement in predictive power with an *R* of 0.282 and an *R*^2^ of 0.080. In this model, along with the previous two models, consumption of sweets (*p* = 0.040) was identified as an additional significant predictor of menstrual pain severity.

In model four, which included the participants' menarche history, further increase in the explained variance was noticed, with *R* reaching 0.354 and *R*^2^ rising to 0.125, with the intensity of menstrual cycle (*p* < 0.001), menstrual cycle regularity (*p* < 0.001, use of pads (*p* = 0.033), and family history (*p* = 0.002) identified as additional significant predictors to overall models (1–4).

In the final model, further improvement in the overall model was observed, with an *R* of 0.504 and an *R*^2^ of 0.254, explaining approximately 19% of the variance in menstrual pain severity. Significant variables included the pain severity (*p* < 0.001), location (*p* < 0.001), duration (*p* < 0.001), and painkiller usage (*p* < 0.001). This model demonstrated that a combination of demographic, behavioral, reproductive, and familial factors collectively contribute to variations in menstrual pain experience.

The ANOVA for the full model confirmed its overall significance (*F* = 2.556, *p* = 0.000), indicating that the predictors jointly explained menstrual pain severity. Collectively, these findings suggest that menstrual pain severity is multifactorial, influenced by demographic characteristics, lifestyle behaviors, reproductive history, and familial predispositions.

## 4 Discussion

The present study examined the prevalence, severity, and associated factors of primary dysmenorrhea among female university students in Jordan, as well as its impact on mental health and academic performance. The results of this study showed a significant prevalence of primary dysmenorrhea among female students at Mutah University, with 49.6%, 46.7%, and 3.7% of students reporting mild, moderate, and severe pain, respectively.

Our findings are consistent with those of a review (14), which indicated a widespread prevalence of dysmenorrhea globally, leading to various consequences, including absenteeism from school or university in many cases, poor academic performance, and reduced participation in school activities at the rates of 20.1%, 40.6%, and 29.6%, respectively. These findings highlight that dysmenorrhea is a widespread problem that impacts performance in various activities and affects academic performance. Importantly, despite the high prevalence of dysmenorrhea, the present study found only a limited association with psychological distress. This may be explained by several contextual and behavioral factors. One possible explanation is that many students may have developed adaptive coping mechanisms over time, leading to increased tolerance and normalization of menstrual pain. In addition, cultural perceptions of menstruation in the local context may contribute to viewing dysmenorrhea as a common and expected experience rather than a condition associated with psychological burden. These factors help explain why psychological distress levels remained relatively low despite frequent pain experiences.

However, it is important to interpret these findings cautiously in the context of the current study, as the observed impact appears to be more related to functional limitations rather than measurable academic performance outcomes. There are potential discrepancies regarding pain and its management, self-reporting, or the availability of effective pain management options. This is evidenced by the fact that the current study found a lower percentage of severe pain related to PD compared with a previous study (14). Approximately 51.6% of the female students in the current study sample use analgesics to alleviate pain associated with PD. Additional evidence for these claims is found in a recent systematic review and meta-analysis (12), which noted the relatively high prevalence of dysmenorrhea among university students and that dysmenorrhea has been shown to have serious consequences for their academic performance. In addition, another global review (11) demonstrated that dysmenorrhea is among the most prevalent gynecological problems reported by female students across the world, affecting more than two-thirds of this demographic. This review highlighted that menstrual problems were significantly linked to psychological stress, lower levels of daily functioning, and lower academic participation.

In the present study, many students missed lectures due to menstrual cramps (43.3%). Most female students participating in the study reported that their daily activities were affected, and primary dysmenorrhea did not significantly predict grade point average (GPA) or formal academic performance. A study consistent with this finding (24) noted that 51.5% of students experienced dysmenorrhea, and that there were associations between primary dysmenorrhea and factors such as chocolate consumption, breakfast habits, positive family history, and menstrual irregularity. These results indicate that mild-to-moderate primary dysmenorrhea may have only a limited effect on formal academic performance, despite affecting attendance and daily functioning.

In contrast, numerous studies conducted in Arab countries have reported strong correlations between primary dysmenorrhea and academic distress. In Saudi Arabia, Dahlawi et al. (10) reported that dysmenorrhea had a significant negative impact on students' academic performance. The study found that 92% of female students had dysmenorrhea and 57.6% of them had moderate to severe pain. It was also found that dysmenorrhea was associated with increased absenteeism, poor concentration, lower academic performance and decreased physical activity. Furthermore, a study conducted in the United Arab Emirates (25) found that 80.5% of female students suffered from dysmenorrhea, with 25.1% experiencing severe pain. More than half of these students missed classes and had difficulty concentrating or studying.

Our findings show that PD has a limited impact on mental health, with DASS scores of (1.11) for depression, (1.15) for anxiety, and (0.82) for stress, indicating relatively mild levels of psychological distress. These findings suggest that primary dysmenorrhea in this cohort is not strongly associated with clinically significant psychological distress. One study (24) partially supports our conclusion, finding no significant associations between PD and mental health. However, it contrasts with the findings of another study (16), which indicated increased psychological distress among female students with dysmenorrhea, with family history and maternal education identified strong predictors of pain and distress severity. Our study found that family history is a strong predictor of pain severity.

Lifestyle habits and sleep duration are important predictors of PD, according to our study. Prolonged sleep and consumption of sweets were strongly associated with increased pain severity. Our findings partially align with a study (17) among Jordanian medical students, which identified lifestyle factors such as sleep and dietary habits as contributors to increased dysmenorrhea severity. In contrast, BMI, smoking, and coffee consumption were not significant predictors in our study. However, another study (26) found a negative correlation between BMI and PD severity.

One important indicator that emerged in our study regarding PD is family history of the condition, with 24.4% of the female students reporting it. Previous studies (16, 27) have shown that familial predisposition is a significant factor associated with the severity of PD. The results of the present study also indicate that older students tend to experience more severe pain, which contradicts the findings from another study (27), where the severity of PD was higher among younger students and those who began puberty early.

The severity of PD was also strongly correlated with menstrual parameters such as age of menarche, regularity, duration of discomfort, and use of medications. It was anticipated that prolonged pain durations or more frequent use of painkillers would result in more severe pain. This is consistent with findings from a study (27) that identified younger age, early menarche, use of analgesics or herbal remedies, and medical visits as important predictors of severe PD. The impact of reproductive history on the severity of PD was further supported by a study (25), which showed that students with earlier menarche frequently experienced moderate-to-severe pain and difficulties performing everyday activities.

Compared to the findings of a study (28) on adolescent girls in Palestinian refugee camps, our study had lower rates of academic disruption, with 48.6% rarely affected, whereas (28) the other study found that 26% missed school, 36% had concentration difficulties, and 39% were unable to complete homework due to PD. Differences in age groups—university students versus teenagers—pain intensity, and supporting environmental factors such as access to healthcare and ease of academic transition may explain these discrepancies.

## 5 Limitations and future recommendations

To the best of our knowledge, this study is the first to examine the association of PD with both mental health and academic performance among female university students in southern Jordan. However, some of the limitations of the present study should be acknowledged. The reliance on a cross-sectional study design and self-reported data may limit the generalizability of the findings and increase the potential for recall bias. In addition, academic performance was measured using self-reported CGPA categories rather than exact continuous GPA values, which may reduce measurement precision and introduce reporting bias. This categorical approach may also attenuate the strength of observed associations between dysmenorrhea and academic performance. Furthermore, the study was conducted in a single university using a convenience sampling technique. This may limit the external validity and generalizability of the findings to other university populations in Jordan and beyond. In addition, the findings should be interpreted within the specific cultural and contextual setting of southern Jordan, which may differ from other regions in terms of health-seeking behavior, menstrual health awareness, and academic environment. Future research should address these limitations in the following manner: Researchers should conduct longitudinal studies, using more structured methods to reduce bias and improve generalizability.

Future research might also benefit from adopting qualitative research approaches to gain deeper insights into the issue. Moreover, future studies will require the use of a control group, as this allows researchers to compare groups of participants and increases the validity of research findings. Interventional studies are also highly desirable for evaluating the efficacy of interventions implemented in reducing primary dysmenorrhea.

## 6 Conclusions

The present study indicates that primary dysmenorrhea is highly prevalent among female university students, with the majority of cases classified as mild to moderate in severity. The findings did not demonstrate a significant association between PD and psychological distress as measured by DASS-21, nor with academic performance as reflected in GPA. However, the results highlight the broader functional burden of dysmenorrhea, particularly its impact on daily activities, attendance, and general discomfort during menstruation. The condition is influenced by multiple factors, including family history, age, sleep patterns, dietary habits, analgesic use, duration of pain, and menstrual characteristics.

Compared to previous studies, more pronounced psychological and academic effects are typically observed in cases of severe dysmenorrhea, suggesting that symptom severity plays a key role in determining overall impact. Therefore, interventions should particularly target students experiencing moderate-to-severe symptoms. At the university level, health education programs, early screening, individualized management strategies, and lifestyle modifications may contribute to reducing the functional burden of dysmenorrhea and improving students' overall wellbeing.

## 7 Implications for practice

The findings of this study carry important implications for university health services and student support systems. Universities are encouraged to strengthen menstrual health education programs to raise awareness of primary dysmenorrhea, its management strategies, and the importance of seeking medical care when necessary. In addition, integrating routine screening for students experiencing moderate-to-severe menstrual pain within university health services may help identify those at risk of functional impairment. Providing accessible counseling, pain management support, and targeted health promotion interventions may contribute to improving students' daily functioning, attendance, and overall wellbeing.

## Data Availability

The raw data supporting the conclusions of this article will be made available by the authors, without undue reservation. The raw data that support the findings of this study are available upon reasonable request.
